# First Reported Human Case of Primary Lymphocutaneous Nocardiosis Caused by *Nocardia shinanonensis*

**DOI:** 10.3390/microorganisms14102346

**Published:** 2026-10-14

**Authors:** Carmen Rodriguez-Cerdeira, Joselyn Yosbeli Ramírez-González, Raúl Vicente Cabezas-Echegoyen, Fernando Martínez-Hernández, Roberto Arenas, Monika Fida, Luary Carolina Martínez-Chavarría, Federico Eliu-Fuentes, Evelyn Cruz-Beltrand, Juan Xicohtencatl-Cortes, Rigoberto Hernández-Castro

**Affiliations:** 1 Fundación Vithas, Grupo Hospitalario Vithas, 28043 Madrid, Spain; 2 Department of Dermatology, Grupo Hospitalario (CMQ-Concheiro), Manuel Olivié 11, 36203 Vigo, Spain; 3 Department of Health Sciences, University of Vigo, Campus of Vigo, As Lagoas, 36310 Vigo, Spain; 4 Centro Dermatológico del Hospital Nacional de Zacamil “Dr. Juan José Fernández”, Mejicanos, San Salvador 01120, El Salvador; joselynyosbeli@gmail.com (J.Y.R.-G.); raulvicentecabezasechegoyen@gmail.com (R.V.C.-E.); fuentesf.md@gmail.com (F.E.-F.); evelyycruz@yahoo.es (E.C.-B.); 5 Departamento de Ecología de Agentes Patógenos, Hospital General Dr. Manuel Gea González, Calzada de Tlalpan 4800, Belisario Domínguez Secc 16, Tlalpan, Ciudad de México 14080, Mexico; fherxyz@yahoo.com; 6 Servicio de Micología, Hospital General “Dr. Manuel Gea González”, Calzada de Tlalpan 4800, Belisario Domínguez Secc 16, Tlalpan, Ciudad de México 14080, Mexico; rarenas98@hotmail.com; 7 Department of Dermatology, Faculty of Medicine, University of Medicine, Rruga e Dibrës No. 371, 1005 Tirana, Albania; monikafida@gmail.com; 8 Departamento de Patología, Facultad de Medicina Veterinaria y Zootecnia, Universidad Nacional Autónoma de Mexico, Circuito exterior S/N. Ciudad Universitaria, Coyoacán, Ciudad de Mexico 04510, Mexico; luary@unam.mx; 9 Laboratorio de Bacteriología Intestinal, Hospital Infantil de Mexico Dr. Federico Gómez., Dr. Márquez 162, Colonia Doctores, Cuauhtémoc, Ciudad de México 06720, Mexico; juanxico@yahoo.com; 10 Departamento de Biología Molecular e Histocompatibilidad, Hospital General “Dr. Manuel Gea González”, Calzada de Tlalpan 4800, Belisario Domínguez Secc 16, Tlalpan, Ciudad de México 14080, Mexico

**Keywords:** *Nocardia shinanonensis*, cutaneous nocardiosis, lymphocutaneous nocardiosis, 16S rRNA sequencing

## Abstract

*Nocardia* species are ubiquitous environmental microorganisms that can cause localized cutaneous, pulmonary or disseminated infections in humans. Primary cutaneous nocardiosis is uncommon and may present as cellulitis, abscesses, ulcers, granulomas or lymphocutaneous disease characterised by nodular lesions distributed along lymphatic pathways. The species most frequently associated with cutaneous nocardiosis is *Nocardia brasiliensis*; however, the number of newly recognised pathogenic species isolated from clinical samples has increased in recent years. This report describes the first reported case of primary lymphocutaneous nocardiosis caused by *Nocardia shinanonensis* in a 36-year-old immunocompetent woman with involvement of the left upper limb. The causative agent was identified by bacteriological culture, matrix-assisted laser desorption/ionisation time-of-flight mass spectrometry (MALDI-TOF), and 16S rRNA gene sequencing. The patient was successfully treated with oral trimethoprim–sulfamethoxazole for 12 weeks, with complete clinical resolution and residual hypertrophic scarring. This case expands the spectrum of human infections caused by *N. shinanonensis* and highlights the importance of molecular tools for accurate species-level identification of rare nocardial pathogens.

## 1. Introduction

*Nocardia* species are aerobic, Gram-positive, partially acid-fast opportunistic pathogens characterised by branching filamentous structures and a beaded appearance. These actinomycetes belong to the order *Mycobacteriales* (formerly *Corynebacteriales*) and are environmental saprophytes widely distributed in soil, dust, decaying organic matter, animal excreta, freshwater, and seawater [1,2]. *Nocardia* spp. can cause localized or disseminated infections, most commonly affecting the lungs, central nervous system, and skin. Infections caused by *Nocardia* species are collectively known as nocardiosis [3]. Nocardiosis is uncommon in immunocompetent individuals but occurs more frequently in patients with impaired cellular or humoral immunity. Nocardiosis predominantly affects immunocompromised patients, including solid organ transplant and hematopoietic stem cell transplant recipients, as well as individuals receiving immunosuppressive therapies or corticosteroids. Nocardiosis in immunocompetent patients is poorly characterized, presenting with clinical heterogeneity such as subacute pneumonia, chronic lung disease, alcohol abuse, type 2 diabetes, cutaneous nodules resembling mycobacterial infections, or disseminated lesions involving cutaneous trauma with muscles and bones involvement, and the central nervous system [4,5,6,7].

Patients with cutaneous nocardiosis frequently report a history of local trauma, such as puncture wounds, abrasions, cat scratches or insect bites, although many do not recall a specific injury [8,9]. The infection may spread to regional lymph nodes, producing a linear chain of nodular lesions along the lymphatic drainage pathway. These lesions often present as ulcerated, draining nodules associated with lymphangitic spread, subcutaneous abscesses and occasionally fever and can mimic other infectious diseases, including sporotrichosis, atypical mycobacterial infections, tuberculosis, syphilis, and leishmaniasis [10,11].

The clinical presentation is usually acute, rapidly progressive, and highly inflammatory [5]. Multifocal lesions generally indicate disseminated disease and are more common in immunocompromised individuals [6,7,8,9]. Primary cutaneous nocardiosis is uncommon, accounting for only 5–7% of all nocardial infections. Such clinical presentation may complicate recognition, leading to delayed diagnosis and treatment [8,10].

Conventional laboratory identification of *Nocardia* is based on the isolation and characterization of the etiological agent from clinical samples. Species-level identification traditionally relies on biochemical testing; however, these methods often lack sufficient sensitivity and specificity required to differentiate clinically relevant *Nocardia* spp. accurately. In addition, biochemical approaches are often time-consuming, resulting in a delayed diagnosis of infection [5].

Molecular methods are essential for rapid and accurate species-level identification of *Nocardia* isolates. Molecular identification has increasingly been adopted in clinical microbiology laboratories. Several molecular markers have been used for this purpose, including the 16S rRNA gene, RNA polymerase β-subunit gene (*rpoB*), heat shock protein gene (*hsp65*), ATPase secretory protein gene (*secA1*) and DNA gyrase subunit B gene (*gyrB*) [12,13,14].

Matrix-assisted laser desorption/ionisation time-of-flight mass spectrometry (MALDI-TOF) has become a useful method for the rapid identification of *Nocardia* and other microorganisms. This technique has revolutionised bacterial identification in clinical microbiology laboratories by providing rapid, simple, cost-effective and reliable species identification [15,16].

The increasing number of clinically recognised *Nocardia* species has improved the understanding of the epidemiology and diversity of nocardial infections [2]. *N. asteroides*, followed by *N. farcinica*, were the most usually identified *Nocardia* species based on phenotypic and biochemical techniques. Nevertheless, major changes have occurred in the taxonomy of the *Nocardia* genus in the last decade, due to the advances in the use of molecular methods. Actually, the species most frequently associated with human disease include *N. nova complex*, *N. farcinica*, *N. transvalensis complex* and *N. brasiliensis*, the latter being the main cause of cutaneous disease in tropical and subtropical regions [1,4,6]. Here, we describe the first reported human case of primary lymphocutaneous nocardiosis caused by *Nocardia shinanonensis* in a 36-year-old woman with involvement of the left upper limb.

## 2. Case Presentation

A 36-year-old woman from San Salvador, El Salvador, with no significant medical history, presented with a 6-week dermatosis that developed after an accidental knife laceration while peeling a tuber. She initially received multiple empirical antibiotic regimens at primary care facilities, including amoxicillin/clavulanate (500/125 mg every 8 h for 10 days), ceftriaxone (1 g daily for 10 days), and clindamycin (600 mg every 6 h for 7 days), without clinical improvement. The lesions progressively spread along the left upper limb and became painful, with purulent drainage, prompting dermatological evaluation.

**Figure 1:**
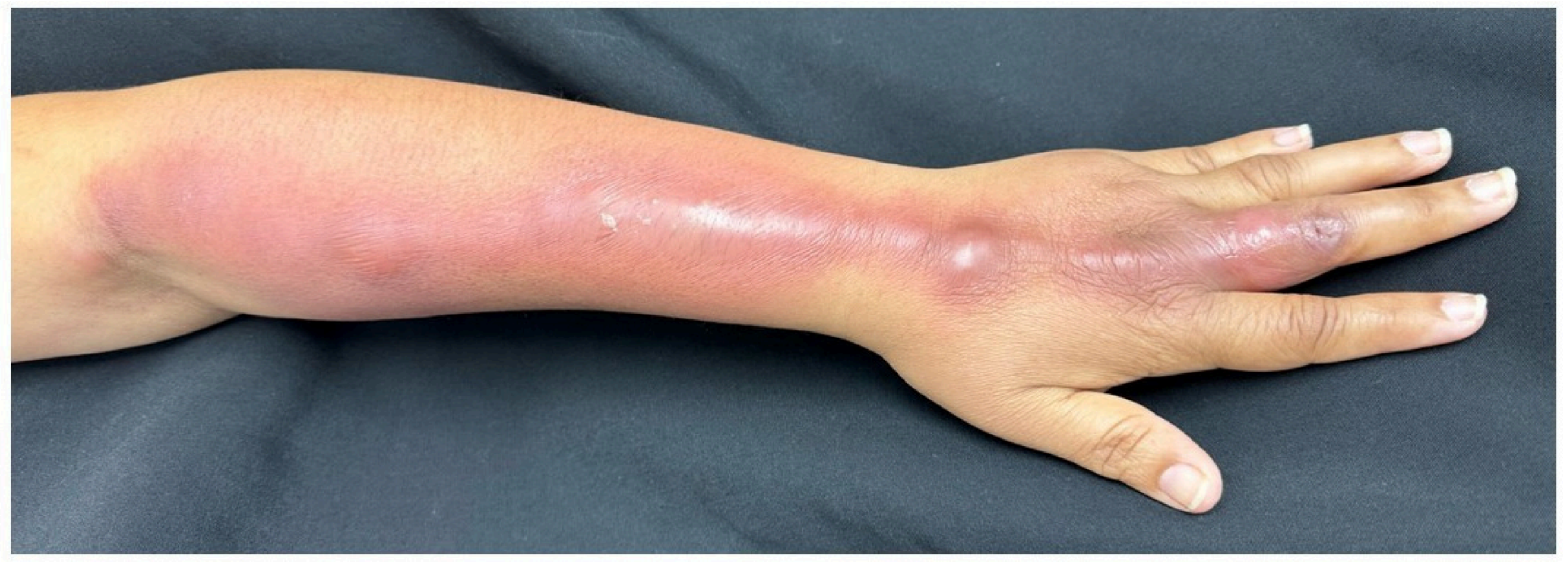
Initial clinical presentation showing nodular lesions distributed linearly along the left upper limb.

Physical examination revealed a dermatosis involving the left upper limb, including the proximal phalanx of the middle finger, dorsum of the hand, anterolateral aspect of the arm, and the axillary region. The lesions consisted of warm nodules distributed linearly along the lymphatic tract, with some abscesses draining seropurulent exudate (Figure 1).

**Figure 2:**
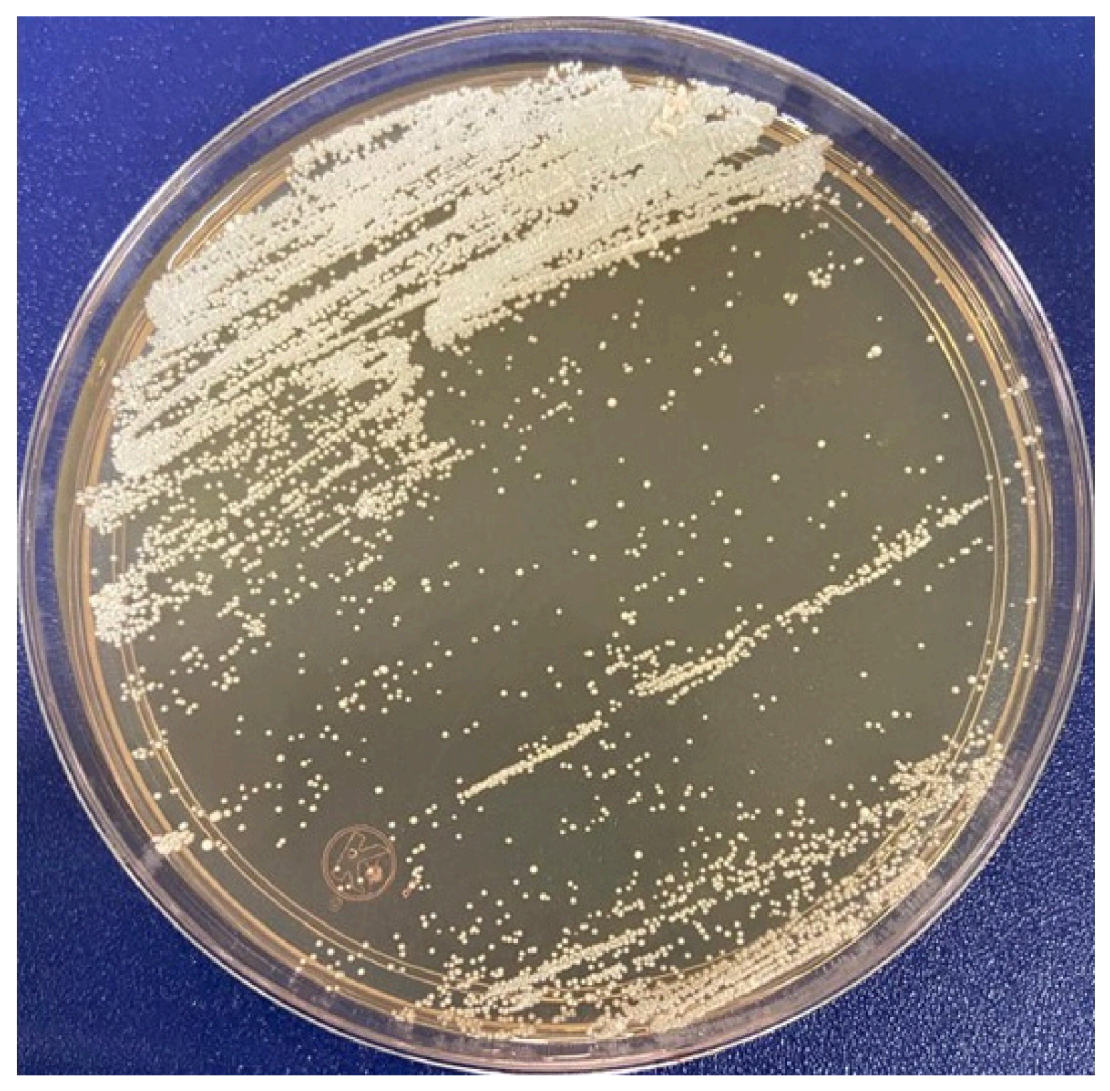
Colony morphology of *N. shinanonensis* after 5 days in Sabouraud agar media.

Lymphocutaneous sporotrichosis was initially suspected, and empirical treatment with oral potassium iodide (1 g every 8 h) was initiated, in this point seropurulent exudate was sent to bacteriological culture. Marked clinical improvement was observed after 10 days of treatment. Bacterial culture was performed on Sabouraud agar under aerobic conditions at 30 °C. After 7 days of incubation, dry, folded and chalky-white colonies were observed, (Figure 2).

**Figure 3:**
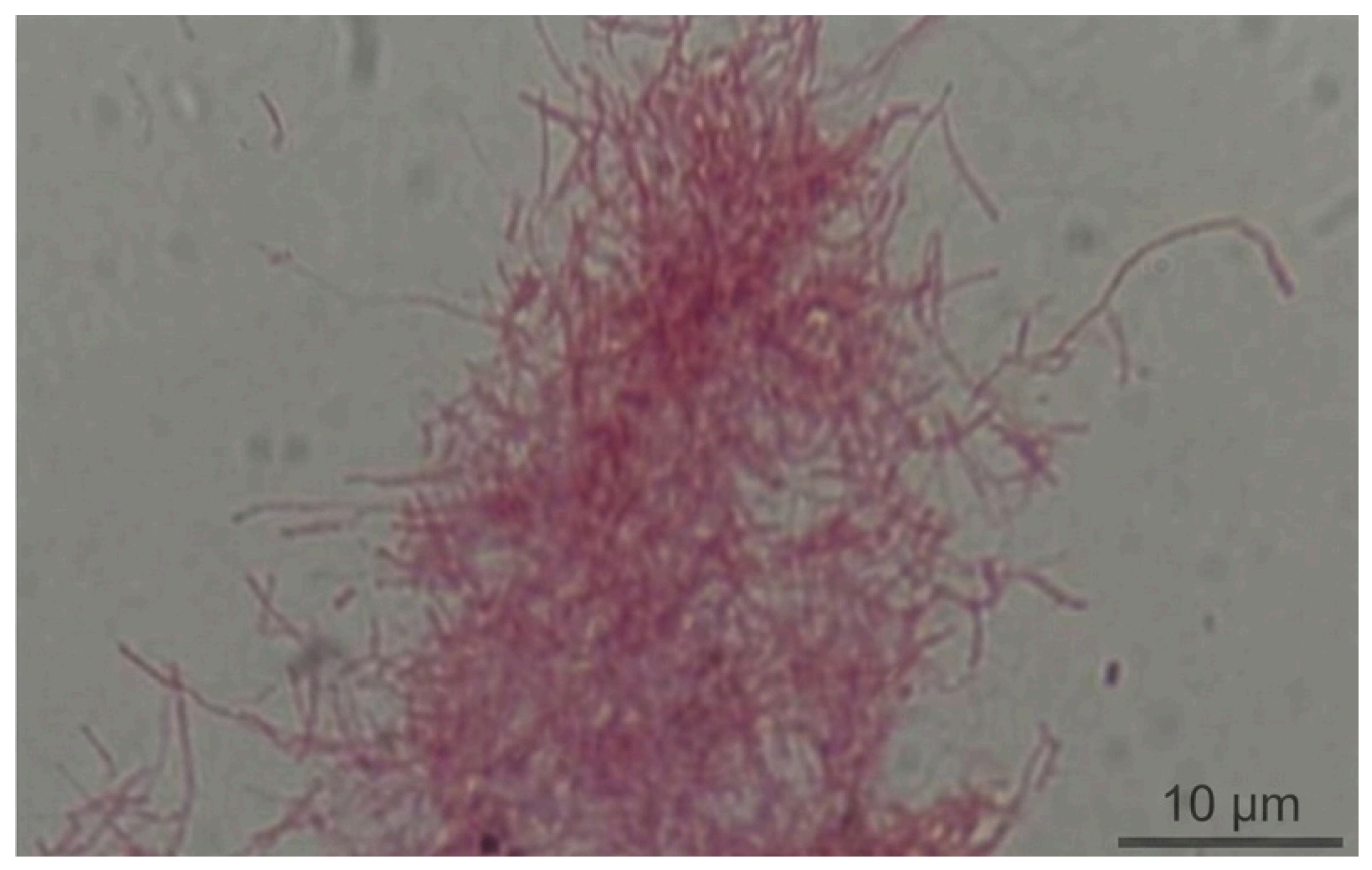
Branching filaments of *N. shinanonensis* from pure culture using a modified Kinyoun acid-fast staining (×1000).

Microscopic examination revealed thin, branching filamentous bacilli that were weakly acid-fast on modified Kinyoun staining (Figure 3). Antimicrobial susceptibility testing was not performed.

Laboratory analysis revealed no anaemia (haemoglobin, 13.2 g dL^–1^) and no leukocytosis (white blood cell count, 9 × 10^3^ cells μL^–1^). The creatinine level was 0.94 mg dL^–1^ (normal range 0.55–1.02 mg dL^–1^). The glucose-6-phosphate dehydrogenase enzyme activity test was within normal limits, as was the lipid profile. A computed tomography scan or X-ray was not performed.

For further species-level identification, amplification and sequencing of the 16S rRNA gene were performed. Briefly, genomic DNA was extracted from pure cultures using the DNeasy Blood and Tissue Kit (Qiagen, Ventura, CA, USA), according to the manufacturer’s instructions. Polymerase chain reaction (PCR) was carried out using the primers 5′-GGATCCTTTTGATCCTGGCTCAGGAC-3′ and 5′-ACTTGACGTCGTCCCCACCTTCCTC-3′, designed based on the 16S rRNA gene sequence of *Nocardia wallacei* ATCC 49,872, formerly *Nocardia asteroides* (accession number AY191251), generating an amplicon of ~1400 bp [17]. The PCR product was purified, and bidirectional sequencing was performed using the fluorescence-based Taq FS Dye Terminator Cycle Sequencing system (Foster City, CA, USA). The resulting sequence was edited using the Vector NTI 11.5 software, and homology analysis was performed using the GenBank database (BLASTn) (https://blast.ncbi.nlm.nih.gov/Blast.cgi, accessed on 23 February 2026), showing 100% identity with *Nocardia shinanonensis* IFM11456 strain (accession number: NR151940). The sequence was deposited in the GenBank database under accession number PZ016551 (*Nocardia shinanonensis* GEA27 strainIn addition, the colony was identified as *N. shinanonensis* using a MALDI-TOF (Bruker MALDI TOF Biotyper) (Billerica, MA, USA). The score value was 2.04 for *N. shinanonensis*, 1.82 with *N. nova* and 1.79 with *N. africana*.

**Figure 4:**
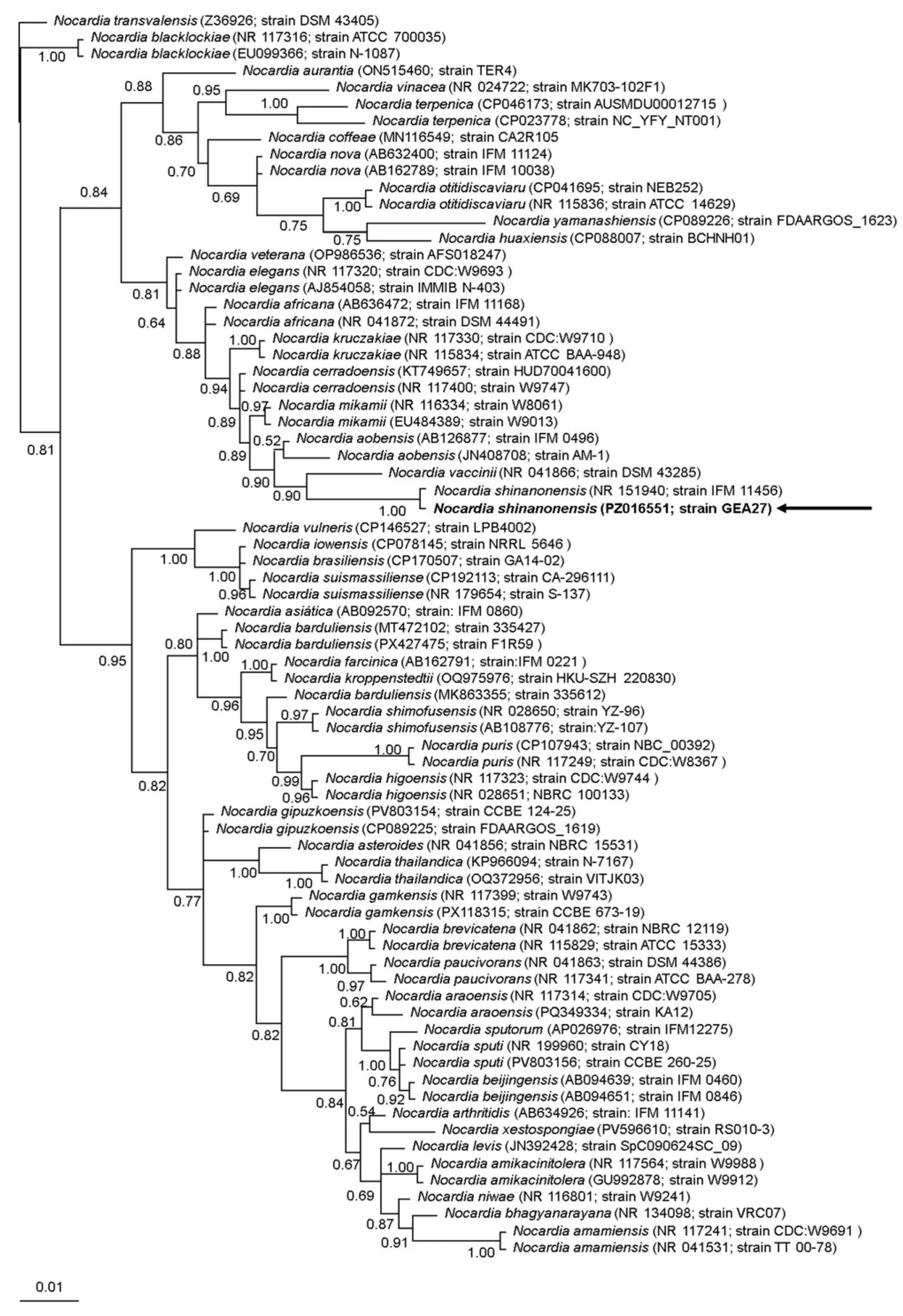
Bayesian phylogenetic tree based on 16S rRNA gene sequences of different *Nocardia* species. Numbers at the nodes indicate posterior probability values. The sequence obtained in the present study is highlighted in bold and indicated by an arrow.

Multiple alignments were performed using the CLUSTALW [18], with manual adjustment in MEGA 5.05 software [19]. The best-fit model of nucleotide substitution was determined using the Akaike Information Criterion in Model test software, version 3.7 [20]. The general time-reversible model with a gamma distribution and invariant sites (GTR + G + I). Phylogenetic reconstruction was performed using Bayesian inference implemented in Mr. Bayes version 3.2.8 [21]. The analysis was run for 4 million generations, sampling trees every 100 generations. Trees generated before reaching stationarity (burn-in period) were discarded, and the remaining trees were used to construct the consensus tree. The phylogenetic analysis, which included our and reference sequences from different *Nocardia* species available in Gen-Bank, showed that the isolate clustered within the *Nocardia shinanonensis* clade with 100% posterior probability (Figure 4).

**Figure 5:**
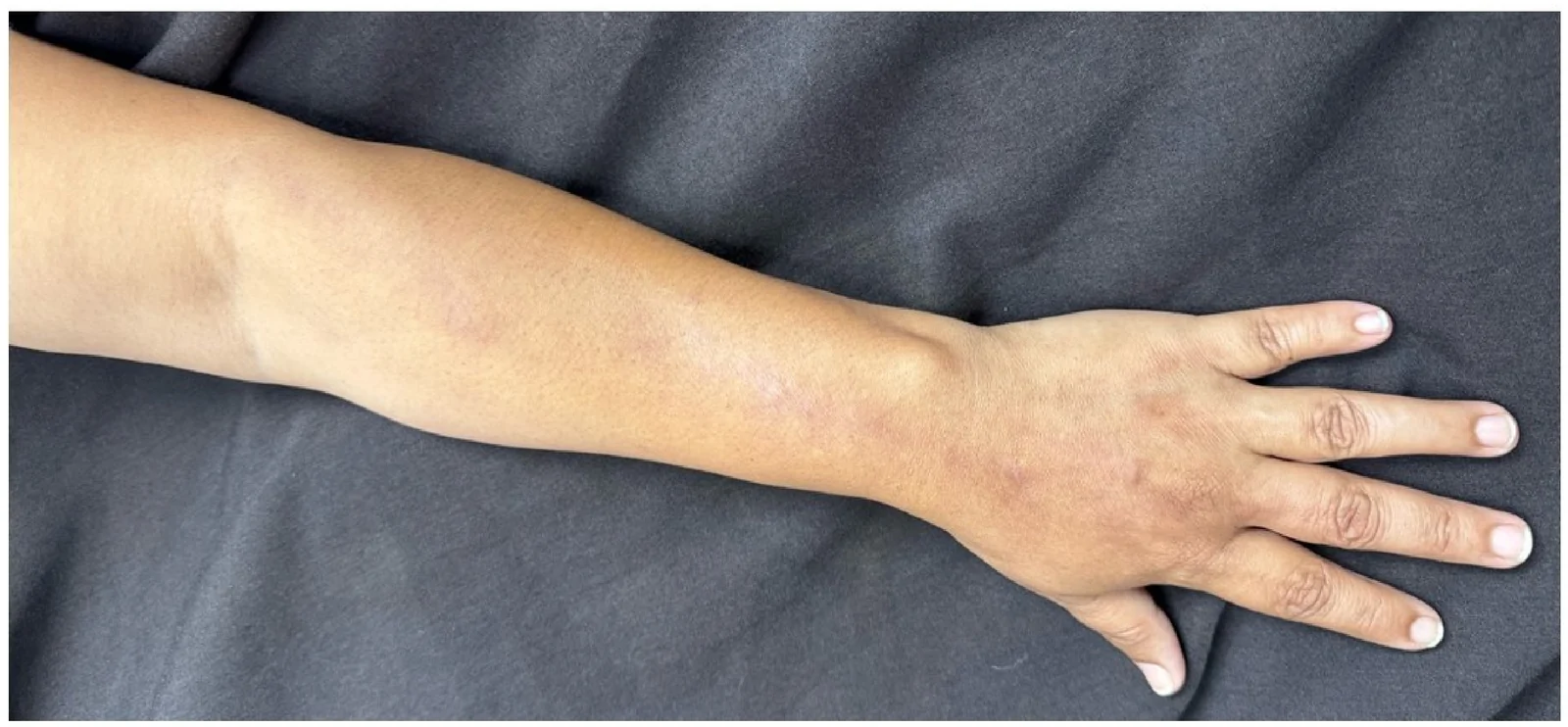
Complete clinical resolution after 12 weeks of antimicrobial therapy, with residual hypertrophic scarring.

Finally, treatment was adjusted to oral trimethoprim–sulfamethoxazole (800/160 mg every 12 h) for 12 weeks. Complete clinical resolution was observed, with only two residual hypertrophic scars remaining (Figure 5).

The clinical course, diagnostic work-up, therapeutic interventions, and outcome are summarized chronologically in Table 1.

## 3. Discussion

Primary cutaneous nocardiosis may present as lymphocutaneous infection, actinomycetoma, superficial skin infection, or secondary cutaneous involvement resulting from hematogenous dissemination [22,23]. This infection usually results from direct traumatic inoculation with soil, plants, or other environmental substrates containing *Nocardia* spp. Our patient presented the classic features of lymphocutaneous nocardiosis, including erythematous nodules distributed linearly along the lymphatic tract, with some abscesses draining seropurulent exudate.

The species most frequently associated with lymphocutaneous nocardiosis is *Nocardia brasiliensis*. However, several other species have been reported in cases of primary cutaneous or lymphocutaneous nocardiosis, such as *N. otitidiscaviarum*, *N. takedensis*, *N. veterana*, *N. vinacea* and *N. pseudobrasiliensis* [24,25,26,27,28]. Here, we describe the first reported human case of lymphocutaneous nocardiosis caused by *Nocardia shinanonensis*. This species was first described in 2016 after being isolated from the aqueous humour of a patient with endophthalmitis in Japan. Its main biochemical characteristics include thermotolerance, growth at temperatures above 45 °C, and the ability to utilise citrate and inositol. In addition, molecular analysis based on the 16S rRNA, *gyrB*, and *secA1* genes demonstrated that this species is closely related to *N. vaccinii*, *N. aobensis*, and *N. makamii* [29]. To our knowledge, this is the first reported human case of lymphocutaneous nocardiosis caused by *Nocardia shinanonensis*.

The genus *Nocardia* currently comprises 131 species, of which at least 54 have been recognised as human pathogens capable of causing a wide range of infections. This places *Nocardia* as the third bacterial genus with the highest number of known pathogenic species [30,31]. Accurate species-level identification is clinically important because different *Nocardia* species vary in geographic distribution, pathogenic potential, and antimicrobial susceptibility profiles [32]. Several methods are available for the identification of *Nocardia* species, including conventional techniques such as direct smear microscopy and culture. The restricted range of available biochemical tests, together with the prolonged incubation time and technical expertise required for interpretation, may delay definitive diagnosis and negatively affect patient management [33].

Molecular methods based on the 16S rRNA gene sequencing have become essential for accurate identification of *Nocardia* species. Other molecular markers, including the housekeeping genes *rpoB*, *hsp65*, *secA1*, and *gyrB*, have also been used individually or in combination in multilocus sequence analysis to improve *Nocardia* species differentiation [12,13,14,15]. Rapid and accurate identification may assist clinicians in selecting appropriate empirical therapy when antimicrobial susceptibility testing is unavailable and may contribute to improved patient management and epidemiological surveillance [1,34]. In the present study, the identification of *Nocardia shinanonensis* was confirmed using a combination of conventional microbiological methods, including bacteriological culture and modified Kinyoun acid-fast staining, together with molecular analysis based on PCR amplification and sequencing of the 16S rRNA gene, as well as MALDI-TOF. This approach allowed accurate species-level identification. Another valuable tool for rapid identification of *Nocardia* species a MALDI-TOF. This technique has emerged as a rapid and reliable approach for the identification of a broad range of microorganisms, mainly through the analysis of ribosomal protein profiles [16,35]. MALDI-TOF is a promising technique that still requires validation, as well as a robust commercial database availability or a combination with in-house database.

Trimethoprim–sulfamethoxazole (TMP-SMX) remains the first-line therapy for nocardiosis. Treatment duration usually ranges from 3–6 months in systemic disease, whereas localised cutaneous forms in immunocompetent patients may respond to shorter courses of 6–12-weeks. Alternative agents, such as amikacin, minocycline, linezolid or imipenem, are generally reserved for cases of sulfonamide intolerance or antimicrobial resistance [5,36,37]. In the present case, an initial diagnosis of lymphocutaneous sporotrichosis led to empirical therapy with oral potassium iodine for 10 days. Certainly, the potassium iodide does not possess direct antimicrobial activity against *Nocardia* spp., in vitro. Instead, its therapeutic efficacy is driven by immunomodulatory and anti-inflammatory pathways. KI imparts an antioxidant effect by directly scavenging reactive oxygen species (ROS). It inhibits ROS formation by inhibiting proinflammatory enzymes like type 2 cyclooxygenase and by inducing overexpression of antioxidant enzymes like superoxide dismutase and catalase, and inhibits reactive nitrogen species formation. Potassium iodide was discontinued when microbiological findings supported molecular confirmation of *N. shinanonensis*, and TMP-SMX was initiated immediately thereafter. There was no overlap between the two treatments. Therefore, complete resolution cannot be unequivocally attributed exclusively to TMP-SMX and may reflect the sequential effect of both treatments. Nevertheless, sustained improvement and complete resolution occurred during subsequent TMP-SMX therapy. This finding highlights nocardiosis as an important differential diagnosis in patients with sporotrichoid lesions.

A major limitation of this case was the absence of antimicrobial susceptibility testing. In our institution, susceptibility testing for microorganisms such as *Nocardia* and *Mycobacterium* is not routinely available in the microbiology laboratory, which may limit therapeutic decision-making in complex cases. In this context, a few laboratories in Mexico perform antimicrobial susceptibility testing; we explored an option near our hospital, but they were unable to conduct the test. In addition, sending the strain outside of Mexico is extremely complicated due to permit requirements and the handling of biological material. There is only one other report of *N. shinanonensis* besides ours, and no antimicrobial susceptibility data are available.

*Nocardia* species were historically divided into groups based on their susceptibility profiles to several antimicrobial families. If the results of antibiotic susceptibility testing are not available, these group antimicrobial susceptibility patterns could be used as a rough guide to determine treatment regimens. Currently, there are only two documented cases of human infection with *N. shinanonensis*; consequently, no treatment guidelines exist. We reviewed the literature for reports on the susceptibility patterns of species phylogenetically related to *N. shinanonensis* (*N. vaccinii* and *N. aobensis*). Nevertheless, antimicrobial susceptibility data are available only for *N. aobensis*, which shows susceptibility to trimethoprim–sulfamethoxazole, linezolid, amikacin, imipenem, ceftriaxone, and clarithromycin [38,39,40]. Based on this information and the therapeutic recommendations of our hospital’s clinicians, the treatment was successful. Furthermore, the characteristics of this microorganism and the effective antimicrobial treatment require further investigation with the accumulation of more clinical cases.

Primary cutaneous nocardiosis represents a diagnostic challenge because it can mimic other granulomatous and lymphangitic infections, particularly sporotrichosis. This case highlights that chronic nodular or ulcerative lesions developing after trauma and failing to respond to conventional antibiotic therapy should raise suspicion for nocardiosis, even in immunocompetent patients.

## 4. Conclusions

The identification of *Nocardia shinanonensis*, a species previously associated only with ocular infection, expands the known clinical spectrum of the genus *Nocardia* and underscores the importance of molecular diagnostic methods, particularly 16S rRNA gene sequencing, for the detection of rare species. The favourable response to trimethoprim–sulfamethoxazole supports its role as first-line therapy while emphasising the importance of individualised treatment based on antimicrobial susceptibility profiles when available. To date, limited information is available regarding *N. shinanonensis* infections, perhaps due to misdiagnosis or the underestimation of human cases, as well as difficulties in clinical and laboratory diagnosis. Reporting emerging pathogens such as *N. shinanonensis* contributes to a better understanding of the taxonomy, epidemiology, antimicrobial susceptibility, and clinical aspects of nocardial infections and may improve diagnostic accuracy in complex dermatologic settings.

## Figures and Tables

**Table 1:** Clinical timeline. Primary lymphocutaneous nocardiosis caused by *Nocardia shinanonensis*.

Time	Clinical Course/Intervention
Day 0	Accidental knife laceration while peeling a tuber.
Following 6 weeks	Progressive nodular lesions along the left upper limb, becoming painful and associated with purulent drainage.
Before dermatology evaluation	Empirical treatment with amoxicillin/clavulanate (500/125 mg every 8 h for 10 days), ceftriaxone (1 g daily for 10 days), and clindamycin (600 mg every 6 h for 7 days), without clinical improvement.
Dermatology evaluation	Lymphocutaneous sporotrichosis was initially suspected and oral potassium iodide (1 g every 8 h) was initiated.
10 days after potassium iodide initiation	Marked clinical improvement was observed.
Microbiological and molecular diagnosis	Culture and modified Kinyoun staining supported Nocardia infection. MALDI-TOF and 16S rRNA gene sequencing showed 100% identity with *Nocardia shinanonensis*.
Definitive treatment	Treatment was adjusted to oral trimethoprim–sulfamethoxazole (800/160 mg every 12 h) for 12 weeks.
Week 12	Complete clinical resolution, with two residual hypertrophic scars.

## Data Availability

The data supporting the findings of this study are available from the corresponding author upon reasonable request. All data supporting the findings of this study are available within the article.
